# Household wealth, marriage, and male fertility in sub-Saharan Africa: a cross-sectional comparative study from three countries

**DOI:** 10.1177/26334941261464394

**Published:** 2026-09-23

**Authors:** Turnwait Otu Michael

**Affiliations:** Department of Sociology, University of Johannesburg, Gauteng, South Africa

**Keywords:** household wealth, male fertility, marital union status, reproductive behaviour, sub-Saharan Africa

## Abstract

**Background:**

Male fertility remains underexplored in demographic and health research across sub-Saharan Africa, where analytical focus is disproportionately placed on women. Yet men’s socioeconomic positions, particularly as reflected by household wealth, and marital union trajectories play critical roles in shaping fertility outcomes and population dynamics.

**Objectives:**

This study examined how household wealth and marital union status intersect to influence fertility outcomes among men in Nigeria, South Africa, and Uganda.

**Design:**

A cross-sectional, comparative analysis was conducted using harmonized data from multiple rounds of the Integrated Public Use Microdata Series–Demographic and Health Surveys (IPUMS-DHS).

**Methods:**

The study analysed a pooled weighted sample of 20,587 men aged 15–59 years. Descriptive statistics, bivariate analyses, and general linear models (GLMs) were used to assess associations between fertility (number of children fathered), household wealth quintiles, marital union status, and their interaction effects.

**Results:**

Mean number of children fathered was highest in Uganda (2.60), followed by Nigeria (2.29), and lowest in South Africa (1.48). Men in multiple marital unions reported substantially higher fertility (mean = 6.10) compared with men in one union (3.44) and never-married men (0.16). Fertility declined across wealth gradients, from 2.57 among the poorest to 1.87 among the richest quintile and increased sharply with age and early union entry. Marital union status was a strong predictor of fertility (Partial η² = 0.082), and the interaction between union status and wealth was statistically significant (*p* < 0.001). Men in one union within the poorest quintile had 1.47 fewer children (*B* = –1.465, *p* < 0.001) compared with men in multiple unions.

**Conclusion:**

Male fertility in sub-Saharan Africa is strongly shaped by the intersection of household wealth and marital union status. Without male-inclusive and socioeconomically responsive reproductive health policies, fertility inequalities and demographic pressures are likely to persist across the region.

## Plain language summary

How men’s wealth and marriage patterns shape the number of children they have in three sub-Saharan African countries (Nigeria, South Africa, and Uganda)

Most research on fertility in sub-Saharan Africa focuses on women, but men also play a central role in deciding when and how many children are born. This study looked at how men’s economic position and marriage histories are linked to the number of children they have fathered in three sub-Saharan African countries: Nigeria, South Africa, and Uganda. It analysed information from more than 20,000 men aged 15–59 years using large national health survey datasets. It compared men who had never married, those married once, and those who had been in more than one marital union, and we examined how their wealth levels influenced fertility. The results showed large differences between countries and across social groups. Men in Uganda had the highest average number of children, while men in South Africa had the lowest. Across all three countries, men who had been in more than one marital union had far more children than those who were married once or never married. Men from poorer households also tended to have more children than men from wealthier households. Importantly, the effect of wealth depended on marriage patterns. For example, poor men who were in only one union had fewer children than men who had multiple unions, showing that marriage history and economic position work together to predict male fertility. These findings highlight the need to include men more fully in family planning and reproductive health programmes. Policies that consider both men’s living conditions and their marriage histories can help reduce inequalities in family size and support healthier families across sub-Saharan Africa.

## Introduction

Male fertility remains a comparatively neglected domain within medical, demographic, and sociological research. This is particularly evident in sub-Saharan Africa (SSA), where reproductive health studies predominantly focus on women.^1,2^ This gendered oversight obscures the pivotal role men play in shaping fertility patterns, union dynamics, and reproductive decision-making. In many African societies, manhood is culturally validated through the ability to marry, father multiple children, and sustain households.^3,4^ This makes paternity not just a biological fact but also a socially, politically, and public health regulated construct. In demographic research, fertility typically refers to reproductive outcomes measured as the number of children born.^1,5^ In the context of men, this study operationalizes fertility as paternity, defined as the number of children ever biologically fathered, reflecting cumulative reproductive outcomes rather than biological fecundity or clinical definitions of infertility.^6,7^

As of 2025, SSA has a population of approximately 1.2 billion people, accounting for 15.9% of the global population and growing at an annual rate of 2.52%.^8^ The region’s total fertility rate (TFR) was 4.3 in 2023; more than double the conventional replacement level of 2.1 children per woman.^9^ Fertility levels vary significantly across countries, from a low TFR of 1.4 in Mauritius to highs of 6.1 in Chad, the Democratic Republic of Congo, and Somalia. Only two countries – Mauritius (1.4) and Cabo Verde (1.5) – have fertility rates below replacement level in SSA. In 2023, Nigeria had a TFR of 4.5, South Africa 2.2, and Uganda 4.3.^9^ These trends emphasis the urgency of understanding male fertility through a critical lens that interrogates structural inequality, prevailing gender norms, and class-based reproductive strategies.

Marriage is central to male reproductive trajectories in the SSA region, particularly given the diversity of marital union forms, including polygyny and union instability.^7,10,11^ While multiple (polygynous) unions may carry stigma in Western contexts, African settings often normalize early and multiple marital unions, especially among men.^12,13^ They consider these as a pathway to social capital and generational continuity.^10,11^ Studies confirm that men in polygynous or serial unions generally report higher fertility than monogamous or never-married counterparts.^7,14^ The affirmation reinforces the intersection of masculinity, union multiplicity, and reproductive output. It also emphasizes that marital patterns, however, are not merely cultural; they are stratified by class and access to economic resources.^15,16^

Household wealth quintile, which refers to a composite measure of a household’s cumulative living standard based on assets, housing characteristics, and access to services, is used in this study as an indicator of socioeconomic status rather than a direct measure of social class.^1,17^ Household wealth and marital union formation are closely linked to male paternity trajectories in SSA, as men with greater economic resources are more likely to enter and sustain marital or nonmarital unions.^7,10^ Wealthier men who can afford bride wealth and support multiple households are more likely to engage in and sustain multiple unions.^18,19^ In contrast, men with fewer economic resources may delay marriage or resort to informal partnerships, impacting their fertility trajectories.^20^ Education further mediates this dynamic, as men with higher educational attainment tend to marry later, adopt modern contraceptive practices, and prefer smaller families.^1,21^

Additionally, fertility among men is shaped by broader socio-political structures and opportunities for mobility.^5,22^ Wealth and education are not only markers of status but also tools through which men negotiate reproductive choices. They sometimes use family size as a means to assert cultural identity or navigate class reproduction.^23,24^ For example, elite men may opt for fewer children with greater investment, while lower-class men may maintain higher fertility norms in the absence of other social capital.^17^

Yet, existing fertility research often fails to analyse these nuances. Many demographic and reproductive health studies treat fertility as a numeric count rather than as an outcome of gendered negotiations, marital union histories, and class-based constraints.^6,25^ Reproductive health inquiry into male fertility must therefore account for the intersectionality of household wealth, marital status, and cultural expectations. This is important, especially in a region where patriarchal norms, structural poverty, and shifting gender roles converge.^26^

This study explores these dynamics by comparing male fertility across Nigeria, South Africa, and Uganda; three countries with divergent cultural, economic, and policy contexts. Nigeria, with entrenched patriarchal systems and high fertility^27^; South Africa, with lower fertility and progressive gender policies^28^; and Uganda, representing a transitional context,^29^ provide a rich comparative landscape. Drawing on harmonized Integrated Public Use Microdata Series–Demographic and Health Surveys (IPUMS-DHS) data,^30^ the study investigates how male fertility is patterned by wealth, education, and marital union status.

In doing so, this study considers male fertility a site where masculine identity, socioeconomic positioning, and reproductive health intersect.^31–33^ The analysis contributes to a growing body of work that repositions men as critical actors in reproductive health and demographic transitions. It also highlights the sociopolitical and public health dimensions of fertility in Africa. This perspective is essential for designing gender-equitable reproductive health policies and addressing fertility-related inequalities across the continent.^34,35^

## Materials and methods

### Study design and data source

This study employed a cross-sectional, secondary data analysis design. It utilized a harmonized male respondent dataset from the IPUMS-DHS. Specifically, the analysis pooled the most recent available data from three SSA countries: Nigeria (2018), South Africa (2016), and Uganda (2016). The study period covered DHS surveys conducted between 2016 and 2018, representing the most recent nationally representative datasets available for the three countries at the time of analysis. Data analysis for this study was conducted between September 2025 and March 2026. IPUMS-DHS provides standardized, recoded DHS variables that facilitate multicountry comparative research.^30^

### Study population and sample size

The analysis focused on men aged 15–59 who were eligible for the DHS men’s questionnaire and had complete responses on key fertility and background variables. After applying sampling weights and handling missing data, the final pooled analytic sample consisted of 20,587 male respondents: 12,104 from Nigeria, 3603 from South Africa, and 4880 from Uganda.

### Exclusion criteria

Sample exclusion criteria included men outside the reproductive age range (under 15 or over 59), those not eligible for the men’s questionnaire, and respondents with missing values on key study variables. To address potential sources of bias, sample weights were applied using DHS men’s weights to ensure national representativeness. Harmonized IPUMS-DHS variables were used to improve cross-country comparability. Although sensitivity analyses were limited, robustness was assessed through the consistency of findings across countries and the stability of model estimates after controlling for multiple covariates. No clinical diagnostic criteria were applicable, as all data were based on self-reported survey responses.

### Inclusion criteria

Men aged 15–59 years who were usual residents or visitors present in the household at the time of the survey, who completed the DHS men’s questionnaire, and who provided complete information on number of children ever fathered, union history, and key covariates were included in the analysis.

### Dependent variable

The outcome of interest was total number of children ever biologically fathered; a continuous count variable derived from the men’s questionnaire. This variable captures cumulative fertility as reported by each male respondent. The inclusion of variables in the analysis was determined by literature and theoretical relevancies and the available of such variables of interest in the datasets.

### Key independent variables

- Union status: Derived from the combination of current marital status and number of unions, categorized as follows: 0 = never married, 1 = one union, and 2 = more than one union.
- Wealth quintile: Based on the DHS wealth index, categorized into five quintiles: poorest, poorer, middle, richer, and richest.
- Education level: Categorized as no education, primary, secondary, and tertiary.
- Age at first union: Recoded into five categories: never married, ⩽24 years, 25–29, 30–34, and 35+ years.
- Current age: Grouped into four age brackets: 15–29, 30–39, 40–49, and 50–59.
- Current work status: Coded as 1 = working, 0 = not working.
- Residence: Urban or Rural, based on DHS classification.
- Family planning (FP) method use: Recoded into no method, traditional method, and modern method.
- Country: Included as a categorical variable to control for cross-national variation.

### Statistical analysis

All analyses were conducted using SPSS version 30, applying the DHS men’s sampling weights to ensure national representativeness. Descriptive statistics (means, percentages) were computed to summarize sample characteristics by country. Bivariate analyses (ANOVA and *t*-tests) assessed associations between fertility and key variables such as union group, education, wealth, occupation, and age. The main analysis employed a general linear model (GLM) to estimate the adjusted effects of union history, wealth quintile, and their interaction on the number of children ever fathered, controlling for education, age at first union, FP method use, current work status, residence, and country. This addresses potential confounders. Age was included as a covariate to account for cumulative exposure to reproductive risk, given that the outcome variable (number of children fathered) is inherently age-dependent. While age-standardized analyses were considered, the inclusion of age categories in the model allows adjustment for life-course accumulation of fertility, consistent with prior DHS-based studies.^6,36,37^ The categorization of union status (never married, one union, multiple unions) does not distinguish between currently married, divorced, separated, or cohabiting men. This aggregation reflects DHS harmonization constraints; however, this limitation is considered in the interpretation of findings.

To assess effect modification, the interaction term between union group and wealth quintile was included to explore whether socioeconomic class moderated the fertility effects of marital experience. To assess the robustness of the interaction between marital union status and household wealth, a cross-tabulation of union group by wealth quintile was conducted (Supplemental Table S1). The distribution of observations across all interaction cells was adequate, with cell sizes ranging from 307 to 2527 respondents. No sparse cells were observed, indicating that the interaction estimates were stable and not driven by small sample bias. To assess multicollinearity, variance inflation factors (VIF) were computed for all independent variables prior to model estimation. All VIF values were below the conventional threshold of 5.^38^ The maximum VIF observed was 2.1, which indicates no serious multicollinearity concerns among the predictors. Variables were also harmonized across countries using IPUMS-DHS standardized labels. All categorical variables were treated as factors in the GLM. Missing values were excluded from the analysis. Model fit was assessed using adjusted *R*² and partial eta squared (η²) to estimate effect sizes. Significance was evaluated at the *p* < 0.05 level, with 95% confidence intervals reported for parameter estimates. STROBE-compliant checklist guided the drafting of the manuscript.^39^

## Results

Table 1 presents the sociodemographic characteristics of 20,587 male respondents from Nigeria, South Africa, and Uganda. The age distribution skewed younger across countries, with 58.9% of Ugandan men aged 15–29, compared to 48.3% in South Africa and 45.0% in Nigeria. Urban residence was highest in Uganda (74.6%), while South Africa had the largest rural share (68.9%). Employment patterns varied sharply. The proportion of men currently working was lowest in South Africa (46.6%), contrasting with 86.0% in Nigeria and 91.7% in Uganda. Educational attainment was also country-specific. Nigeria had the highest share of men with no education (20.1%), while 71.8% of South African men had secondary education. Regarding household wealth, the distribution was relatively balanced across countries, although South Africa had a slightly higher proportion in the bottom two quintiles.

**Table 1.** Sample characteristics of male respondents by country.

Variable	Nigeria (*n* = 12,104) (%)	South Africa (*n* = 3,603 (%)	Uganda (*n* = 4,880) (%)	Total (*N* = 20,587) (%)
Age				
15–29	5442 (45.0)	1739 (48.3)	2875 (58.9)	10056 (48.8)
30–39	3301 (27.3)	839 (23.3)	1093 (22.4)	5233 (25.4)
40–49	2261 (18.7)	609 (16.9)	675 (13.8)	3545 (17.2)
50–59	1100 (9.1)	415 (11.5)	237 (4.9)	1752 (8.5)
Residence				
Rural	5862 (48.4)	2484 (68.9)	1240 (25.4)	9586 (46.6)
Urban	6242 (51.6)	1119 (31.1)	3640 (74.6)	11,001 (53.4)
Currently working				
Not working	1696 (14.0)	1923 (53.4)	405 (8.3)	4024 (19.5)
Working	10,408 (86.0)	1680 (46.6)	4475 (91.7)	16,563 (80.5)
Education level				
No education	2430 (20.1)	108 (3.0)	202 (4.1)	2740 (13.3)
Primary	1668 (13.8)	511 (14.2)	2679 (54.9)	4858 (23.6)
Secondary	5842 (48.3)	2588 (71.8)	1380 (28.3)	9810 (47.6)
Tertiary	2165 (17.9)	397 (11.0)	619 (12.7)	3181 (15.4)
Wealth quintile				
Poorest	1945 (16.1)	696 (19.3)	805 (16.5)	3446 (16.7)
Second	2046 (16.9)	740 (20.5)	853 (17.5)	3639 (17.7)
Middle	2383 (19.7)	777 (21.6)	967 (19.8)	4127 (20.0)
Fourth	2731 (22.6)	743 (20.6)	1095 (22.4)	4569 (22.2)
Richest	2999 (24.8)	647 (18.0)	1159 (23.8)	4805 (23.3)
Union status				
Never married	4963 (41.0)	2143 (59.5)	2047 (41.9)	9153 (44.5)
One union	6309 (52.1)	1278 (35.5)	1952 (40.0)	9539 (46.3)
More than one union	832 (6.9)	183 (5.1)	881 (18.1)	1896 (9.2%)
Age at first union (mean, SD)	26.76 (6.1)	27.06 (7.3)	22.69 (4.9)	25.79 (6.3)
No. of women fathered children with				
None	5347 (44.2)	1662 (46.1)	2146 (44.0)	9155 (44.5)
One woman	5988 (49.5)	1249 (34.7)	1779 (36.5)	9016 (43.8)
More than one	769 (6.4)	693 (19.2)	955 (19.6)	2417 (11.7)
Current FP methods use				
No method	10,053 (83.0)	1690 (46.9)	2953 (60.5)	14,696 (71.4)
Traditional	236 (1.9)	4 (0.1)	190 (3.9)	430 (2.1)
Modern	1816 (15.0)	1909 (53.0)	1738 (35.6)	5463 (26.5)
Occupation type				
Not currently working	1460 (12.1)	1713 (47.5)	281 (5.8)	3454 (16.8)
Professional/Technical	1459 (12.1)	289 (8.0)	491 (10.1)	2239 (10.9)
Clerical/Sales/Services	1920 (15.9)	80 (2.2)	208 (4.3)	2208 (10.7)
Agriculture	4202 (34.7)	97 (2.7)	2052 (42.1)	6351 (30.8)
Household/Domestic services	1073 (8.9)	255 (7.1)	459 (9.4)	1787 (8.7)
Skilled/Unskilled manual/Other	1990 (16.4)	1170 (32.5)	1388 (28.4)	4548 (22.1)

Sample/percentages were weighted using DHS men’s sample weights.

FP, family planning.

Union status revealed that never-married men were most common in South Africa (59.5%), while Uganda had the highest share of men with more than one union (18.1%). The average age at first union was lowest in Uganda (22.7 years) and highest in South Africa (27.1 years). Fertility patterns showed that a majority of men across all countries had fathered children with one woman, but multiple-partner fertility was highest in Uganda (19.6%) and South Africa (19.2%). Contraceptive use differed substantially. Modern method use was highest in South Africa (53.0%), while Nigeria had the lowest modern use (15.0%) and highest non-use (83.0%). Occupational profiles reflected labour market and rural-urban dynamics. Agriculture dominated in Uganda (42.1%) and Nigeria (34.7%), while manual labour and non-working status were more prevalent in South Africa. Notably, South Africa had the highest share of unemployed men (47.5%) and the lowest share in agriculture (2.7%).

Figure 1 displays the average number of children ever fathered by men across four age groups in Nigeria, South Africa, and Uganda. As expected, mean fertility increased steadily with age in all three countries, reflecting a life course accumulation of childbearing. At ages 15–29, average fertility remained low, ranging from 0.28 children in Nigeria to 0.62 in Uganda, indicating limited early paternity. By ages 30–39, mean fertility rose sharply, with men in Uganda reporting the highest average of 4.00 children, nearly double that of their South African counterparts (1.84 children). The gap widened further in older age groups. Among men aged 50–59, Ugandan men had fathered 8.10 children on average, compared to 6.64 in Nigeria and 3.74 in South Africa. Across all age groups, South African men reported the lowest fertility, while Ugandan men had the highest, suggesting differences in fertility norms, marriage timing, and contraceptive behaviour. The overall mean number of children fathered was 2.60 in Uganda, 2.29 in Nigeria, and 1.48 in South Africa. These highlight cross-national and age-related disparities in male fertility. It also reinforces the influence of socio-cultural and demographic dynamics on men’s reproductive trajectories.

**Figure 1.**
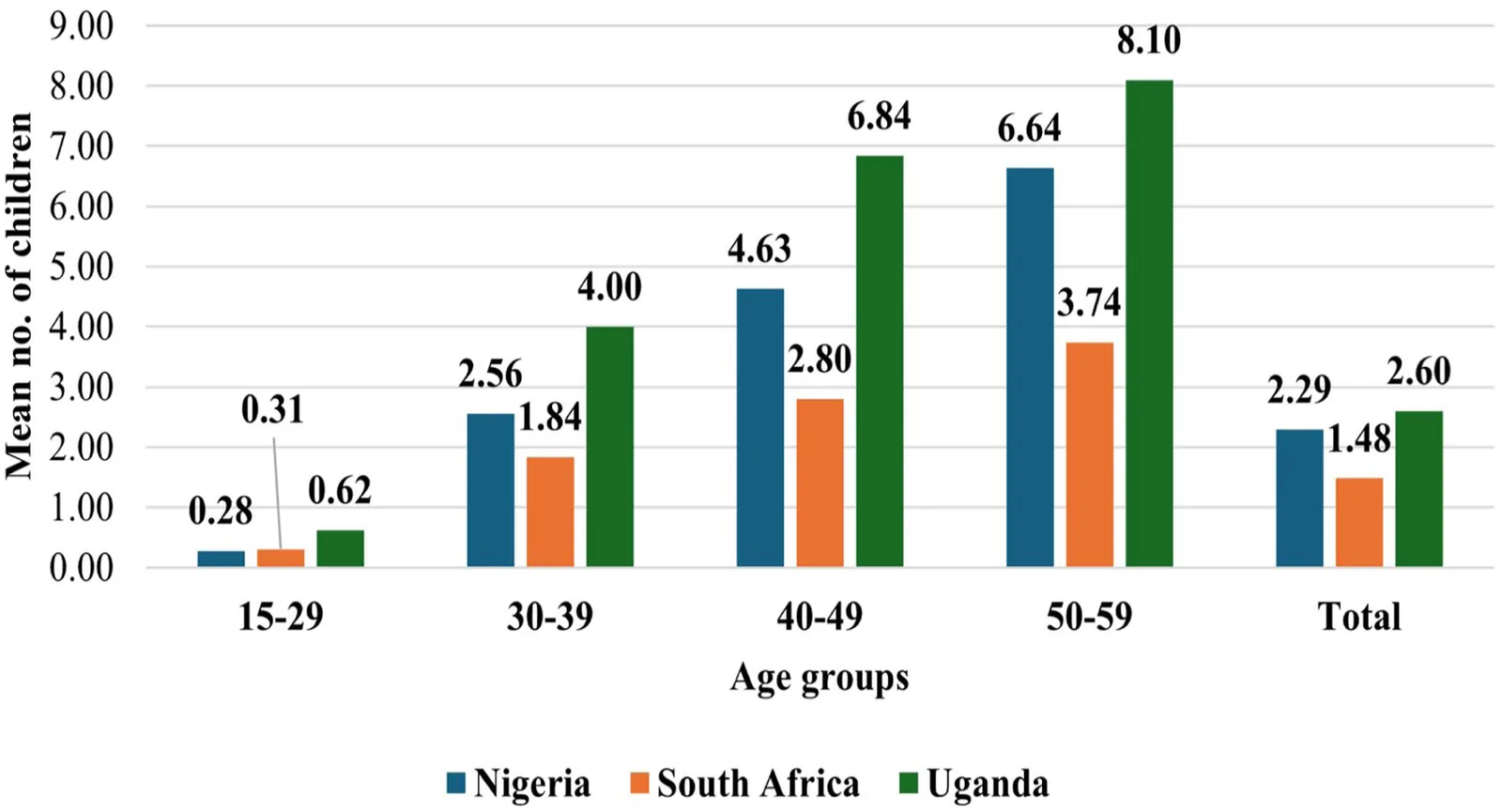
Mean number of children ever biologically fathered across age groups.

Figure 2 shows the mean number of children fathered by men in relation to their age at first union across Nigeria, South Africa, and Uganda. As expected, never-married men reported the fewest children, ranging from 0.02 in Nigeria to 0.56 in South Africa. This reflects limited paternity outside of formal unions. Among men who had entered a union, those who married at age 24 or younger had the highest fertility in all three countries: 4.58 in Uganda, 4.39 in Nigeria, and 2.76 in South Africa. This supports the well-established pattern that early union formation is strongly associated with higher lifetime fertility. Interestingly, in Nigeria and Uganda, mean fertility declined gradually with increasing age at first union. However, in South Africa, fertility was lowest among men who married between ages 25–29 and 30–34, but rose slightly again among those who married at age 35 or older (mean = 3.61). This indicates possible compensatory or delayed fertility behaviour. The results also highlight the fertility-reinforcing effect of early unions, particularly in Uganda and Nigeria, while also suggesting country-specific patterns of late fatherhood in South Africa.

**Figure 2.**
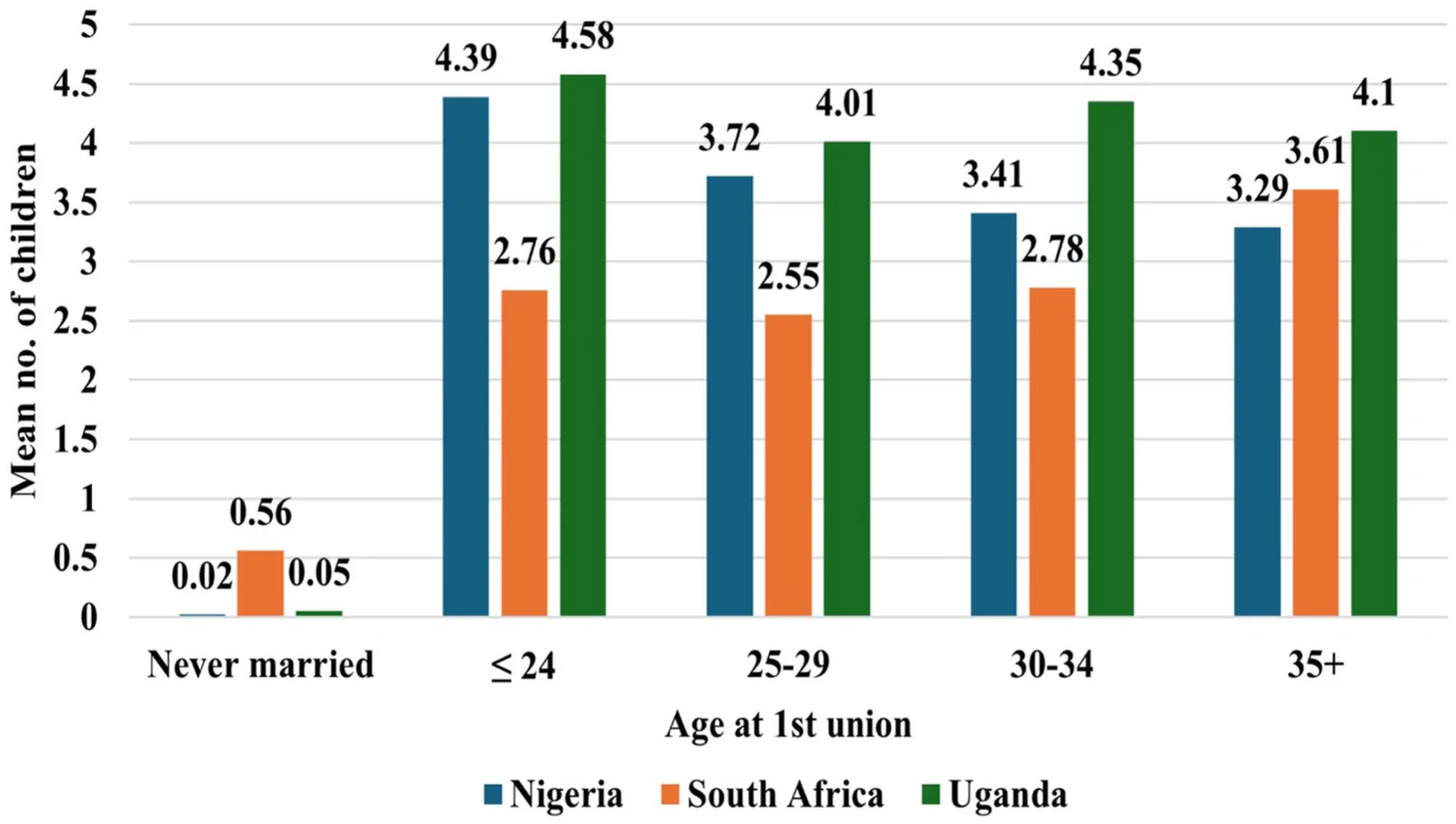
Mean number of children ever biologically fathered across age at first union.

Table 2 presents the bivariate associations between key sociodemographic characteristics and the number of children ever fathered among 20,587 men across Nigeria, South Africa, and Uganda. Across all variables, differences in mean fertility were statistically significant (*p* < 0.001). Union group was the strongest differentiator of fertility outcomes: men in more than one union reported the highest mean number of children (6.10), compared to those in one union (3.44) and the never married (0.16). Educational attainment showed an inverse relationship with fertility. Men with no education had the highest fertility (mean = 3.30), while those with secondary or tertiary education reported substantially fewer children (mean = 1.62 and 1.99, respectively). Fertility also decreased progressively with household wealth status. Men in the poorest quintile had a mean of 2.57 children, while those in the richest quintile reported 1.87. Similarly, men engaged in agricultural occupations had higher fertility (mean = 2.99) compared to professionals and clerical workers.

**Table 2.** Bivariate association between household wealth, marital union status, and number of children ever fathered (pooled data, *N* = 20,587).

Variable	Weighted (*N* = 20,587)	Mean children fathered	Std. deviation	*p*-Value	Test used
Marital union status				<0.001	ANOVA
Never married	9153	0.16	0.64		
One union	9539	3.44	2.63		
More than one union	1896	6.10	4.16		
Education level				<0.001	ANOVA
No education	2740	3.30	4.01		
Primary	4858	2.99	3.4		
Secondary	9810	1.62	2.3		
Tertiary	3181	1.99	2.5		
Wealth quintile				<0.001	ANOVA
Poorest	3446	2.57	3.54		
Second	3639	2.42	3.22		
Middle	4127	2.32	3.10		
Fourth	4569	2.09	2.87		
Richest	4805	1.87	2.26		
Occupation				<0.001	ANOVA
Not currently working	3454	0.63	1.68		
Professional/Technical	2239	2.50	2.62		
Clerical/Sales/Services	2208	2.36	2.94		
Agriculture	6351	2.99	3.65		
Household/Domestic services	1787	2.05	2.69		
Skilled/Unskilled manual/Other	4548	2.24	2.55		
Currently working				<0.001	*t*-test
Not working	4024	0.68	1.77		
Working	16,564	2.60	3.10		
Residence				<0.001	*t*-test
Urban	9586	1.93	2.49		
Rural	11,001	2.48	3.34		
Current age				<0.001	ANOVA
15–29	10,056	0.38	0.94		
30–39	5233	2.75	2.24		
40–49	3545	4.74	3.13		
50–59	1752	6.15	4.09		
Age at first union					
Never married	9153	0.16	0.64	<0.001	ANOVA
⩽24	5369	4.28	3.34		
25–29	3194	3.61	2.82		
30–34	1757	3.42	2.74		
35+	1115	3.41	2.97		
Current FP methods use				<0.001	ANOVA
No method	14,696	2.32	3.09		
Traditional	430	3.43	2.95		
Modern	5462	1.88	2.63		
Country				<0.001	ANOVA
Nigeria	12,104	2.29	3.03		
South Africa	3603	1.48	2.03		
Uganda	4880	2.60	3.38		

Mean differences were computed using weighted data; ANOVA was used for variables with 3+ categories; independent *t*-tests for binary comparisons; *p*-values were derived from *F*-tests or *t*-tests depending on variable type.

FP, family planning.

Employment status was a major factor. Currently working men had significantly higher fertility (mean = 2.60) than those not working (mean = 0.68). Rural residents also reported higher fertility (mean = 2.48) than their urban counterparts (mean = 1.93). As expected, fertility increased with age. Men aged 15–29 had very low fertility (mean = 0.38), while those aged 50–59 had the highest (mean = 6.15). Early age at first union was also linked to higher fertility with men who entered union at or before age 24 reporting a mean of 4.28 children. Use of modern FP methods was associated with lower fertility (mean = 1.88), while those using no method or traditional methods reported more children. Cross-country differences were evident. Ugandan men had the highest average number of children fathered (2.60), followed by Nigerian men (2.29), while South African men reported the lowest (1.48). This demonstrates the demographic and cultural diversity across countries.

As shown in Table 3, the GLM assessed the influence of union group, wealth quintile, and their interaction on the number of children ever fathered by men, controlling for education, age at first union, FP method use, work status, residence, and country. The overall model was statistically significant, *F*(26, 20,587) = 722.49, *p* < 0.001, and explained 47.0% of the variance in male fertility (adjusted *R*² = 0.469). Partial η² also known as partial eta squared (η²) is a measure of effect size. It tells us the proportion of variance in the dependent variable that is explained by a specific predictor, after accounting for all other variables in the model. From the results, union history was a strong predictor of male fertility. Compared to men in more than one union, those who were currently in one union had 1.80 fewer children on average (*B* = −1.801, *p* < 0.001), while never-married men had 4.40 fewer children (*B* = −4.398, *p* < 0.001). This confirms the expected fertility gradient by marital experience, with polygynous or serially partnered men reporting the highest reproductive output.

**Table 3.** General linear model parameter estimates for number of children ever fathered (pooled data, *N* = 20,587).

Predictor	*B*	SE	95% CI	*p*-Value	Partial η²
Union status (ref: more than one union)				<0.001	0.082
Never married	−4.398	0.148	[−4.689, −4.107]	<0.001	0.040
One union	−1.801	0.132	[−2.060, −1.542]	<0.001	0.009
Wealth quintile (ref: Richest)				<0.001	0.004
Poorest	+1.868	0.165	[1.544, 2.192]	<0.001	0.006
Poorer	+1.057	0.165	[0.733, 1.380]	<0.001	0.002
Middle	+1.053	0.167	[0.726, 1.379]	<0.001	0.002
Richer	+0.867	0.172	[0.530, 1.203]	<0.001	0.001
Union × wealth quintile interaction				<0.001	0.009
Never married × Poorest	−2.218	0.177	[−2.565, −1.870]	<0.001	0.007
Never married × Poorer	−1.194	0.177	[−1.541, −0.847]	<0.001	0.002
Never married × Middle	−1.101	0.179	[−1.452, −0.751]	<0.001	0.002
Never married × Richer	−0.933	0.184	[−1.294, −0.572]	<0.001	0.001
One union × Poorest	−1.465	0.177	[−1.812, −1.118]	<0.001	0.003
One union × Poorer	−0.615	0.176	[−0.961, −0.270]	<0.001	0.001
One union × Middle	−0.525	0.178	[−0.873, −0.176]	0.003	0.001
One union × Richer	−0.553	0.182	[−0.911, −0.196]	0.002	0.001
Education (ref: Higher)				<0.001	0.014
No education	+0.841	0.065	[0.713, 0.969]	<0.001	0.008
Primary	+0.617	0.056	[0.508, 0.726]	<0.001	0.006
Secondary	+0.096	0.046	[0.005, 0.187]	0.038	0.001
Age at first union (ref: 35+)				<0.001	0.002
⩽24 years	+0.366	0.073	[0.222, 0.509]	<0.001	0.001
25–29 years	+0.114	0.075	[−0.033, 0.262]	0.129	0.001
30–34 years	+0.099	0.083	[−0.063, 0.261]	0.230	0.001
Family planning use (ref: Modern)				<0.001	0.001
No method	−0.086	0.037	[−0.159, −0.013]	0.020	0.001
Traditional method	+0.357	0.110	[0.142, 0.571]	0.001	0.001
Current work status (ref: Yes)	−0.001	0.045	[−0.089, 0.087]	0.985	0.001
Urban–rural residence (ref: Rural)	−0.097	0.037	[−0.169, −0.025]	0.009	0.001
Country (ref: Uganda)				<0.001	0.001
Nigeria	+0.170	0.043	[0.086, 0.255]	<0.001	0.001
South Africa	+0.113	0.058	[0.000, 0.226]	0.051	0.001
Model summary: *F*(26, 20587) = 722.49, *p* < 0.001; *R*² = 0.470, adjusted *R*² = 0.469					

Weighted analysis using men’s sample weight; *N* = 20,587; Partial η² = effect size; Significant at *p* < .05.

Compared to men in the richest quintile, those in poorer wealth groups had significantly higher fertility. Men in the poorest quintile reported an average of 1.87 more children (*B* = 1.868, *p* < 0.001), while those in the middle and richer categories also had significantly higher fertility (*B* range: 0.87–1.05, all *p* < 0.001). These results suggest an inverse relationship between socioeconomic status and fertility. A significant interaction was found between union group and wealth quintile (*p* < 0.001), indicating that the fertility difference across union groups varied by class. For instance, never-married men in the poorest quintile reported 2.22 fewer children than their more-than-one-union counterparts in the same quintile (*B* = −2.218, *p* < 0.001). Similarly, men in one union and the poorest group reported 1.47 fewer children (*B* = −1.465, *p* < 0.001) than the multi-union reference group. This pattern shows that the fertility-reducing effect of wealth is most pronounced among men in fewer or no unions, while multi-union men maintain high fertility regardless of wealth.

Educational attainment was inversely associated with fertility. Compared to men with higher education, those with no education had 0.84 more children (*B* = 0.841, *p* < 0.001), and those with primary and secondary education also had significantly higher fertility (*B* = 0.617 and 0.096, respectively). Men who entered a union at age 24 or younger had significantly more children than those who married later (*B* = 0.366, *p* < 0.001). The effect for ages 25–29 and 30–34 was not significant. Compared to users of modern contraception, men who used no method reported slightly fewer children (*B* = −0.086, *p* = 0.020), while those using traditional methods had higher fertility (*B* = 0.357, *p* = 0.001). This may reflect reverse causality, where men with larger families resort to traditional FP due to unmet need. Men residing in urban areas had fewer children on average than rural men (*B* = −0.097, *p* = 0.009). This is consistent with global fertility trends linked to urbanization and delayed childbearing. Country-level differences were modest but significant. Compared to Uganda, men in Nigeria reported slightly higher fertility (*B* = 0.170, *p* < 0.001), while those in South Africa also had higher fertility, though marginally (*B* = 0.113, *p* = 0.051).

Figure 3 shows that men in more than one union consistently exhibit the highest predicted fertility across all wealth quintiles, with relatively modest variation by wealth. In contrast, fertility declines with increasing wealth among men in one union, while remaining uniformly low among never-married men. This pattern indicates that the association between socioeconomic status and male fertility is conditional on union experience, with union status moderating the male fertility across wealth groups. The predicted values were derived from a GLM including union status, wealth quintile, and their interaction.

**Figure 3.**
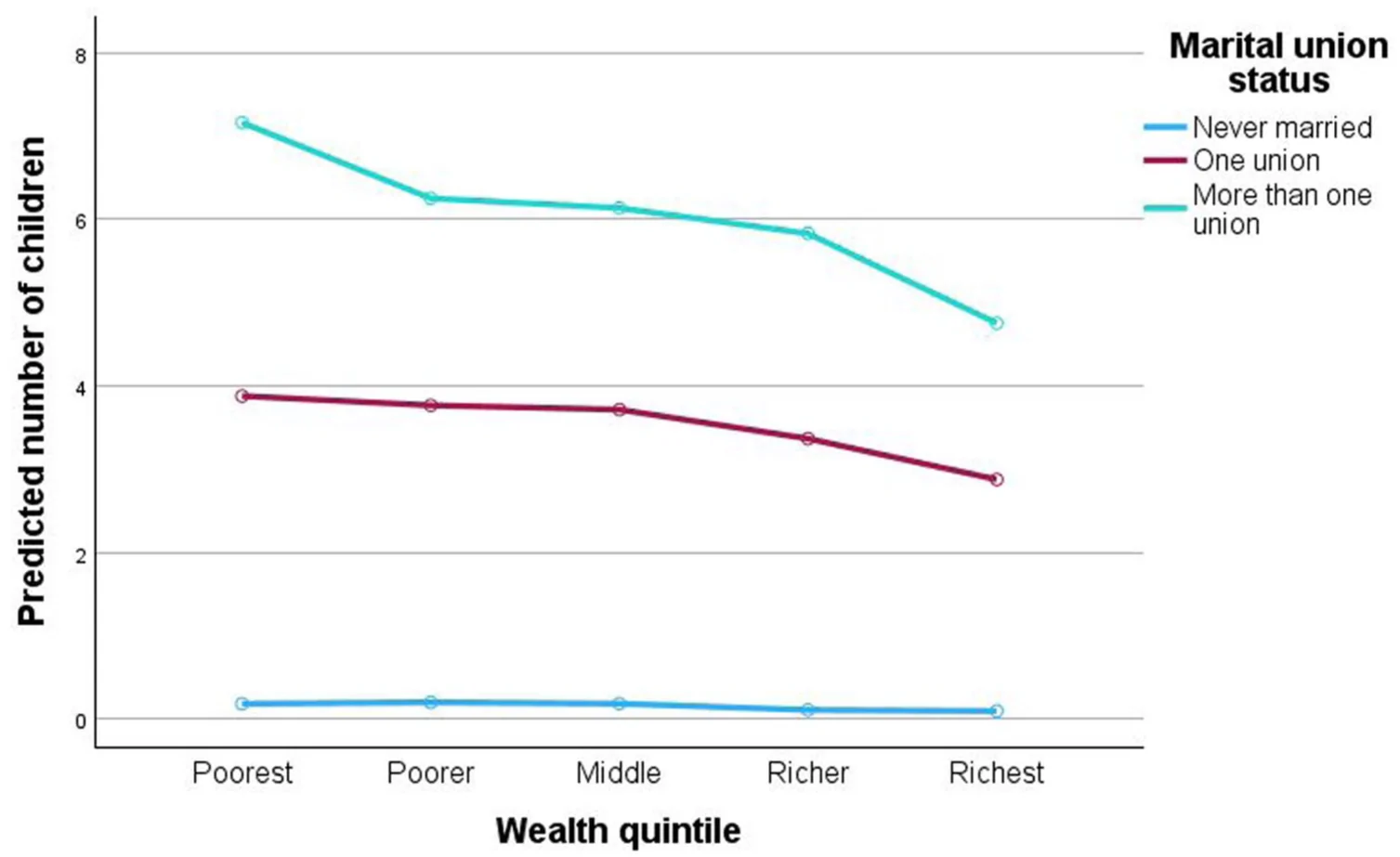
Predicted number of children ever fathered by marital union status across wealth quintiles.

## Discussion

This study provides an intersectional analysis of male fertility patterns in three diverse SSA countries (Nigeria, South Africa, and Uganda). It focuses on how household wealth, marital status, and their interaction influence reproductive health outcomes. The findings affirm classical demographic theories while introducing new perspectives on male reproductive trajectories within patriarchal contexts.^40,41^ Marital union emerged as the most potent predictor of male fertility, with men in multiple unions reporting significantly higher numbers of children compared to those in one union or never married. This aligns with the polygyny–fertility hypothesis, which has been documented in the region.^4,10^ The finding also resonates with cultural expectations where masculine identity is affirmed through fatherhood and the capacity to sustain multiple partnerships.^25,35^

Household wealth showed an inverse association with fertility, consistent with demographic transition theory.^42^ As in prior research, wealthier men tended to have fewer children, likely reflecting higher opportunity costs of childrearing, investment in child quality, and greater access to contraceptive knowledge.^23,43^ However, the interaction analysis nuances this relationship: among never-married or single-union men, fertility declined with increasing wealth, but among men in multiple unions, fertility remained high across all wealth quintiles. This echoes that high fertility persists in patriarchal social systems despite economic gains, especially among men who command marital opportunities.^37^ This interaction departs from linear models and supports newer frameworks that stress how reproductive behaviour is co-constructed by material resources and gendered social norms.^34,44^ It suggests the presence of a ‘patriarchal fertility privilege’, wherein high-status men can maintain high fertility regardless of economic incentives to limit family size.

Building on this, the findings can be more fully interpreted through the lens of ‘patriarchal fertility privilege’, defined as the socially embedded advantages that enable men to achieve higher cumulative fertility through multiple partnerships and union trajectories. In this context, men possess greater social and relational power to initiate, sustain, and reproduce across multiple unions, often with fewer social or biological constraints than women.^45,46^ This privilege is reinforced by gendered norms, economic structures, and marital systems such as polygyny, which collectively expand men’s opportunities for cumulative paternity.^4,10^ In this study, men in multiple unions maintained consistently high fertility across all wealth quintiles, indicating that reproductive outcomes are not solely constrained by socioeconomic resources but are also shaped by rooted gendered power relations. In many SSA contexts, the cultural acceptance of polygyny, lower social sanctions for multiple partnerships, and men’s relatively greater control over union formation further consolidate this privilege.^10,46,47^ This perspective helps explain why, even among lower wealth groups, men in multiple unions are able to sustain high levels of paternity, emphasizing the structural nature of male reproductive advantage.

Educational attainment had a clear inverse relationship with fertility. Men with no or primary education reported more children than those with tertiary education. This supports earlier findings linking higher education to delayed marriage, increased contraceptive use, and lower fertility intentions.^36,48^ Interestingly, secondary education was also associated with significantly reduced fertility. With this, education reinforces its critical role in shaping male reproductive health behaviour. Early age at first union was associated with higher fertility. This finding confirms that longer exposure to unions increases the opportunity for childbearing.^11,49^ However, the lack of significant fertility difference among those who entered unions at older ages suggests a possible compression of childbearing into a shorter period, especially among men with multiple unions.

FP use showed mixed patterns. Men using traditional methods had higher fertility, possibly indicating post-hoc adoption after large family sizes. This supports the observation that male engagement in FP is often reactive and shaped by cultural norms or access constraints.^24,50^ Urban residence was associated with lower fertility. This was in line with global fertility transitions and African DHS trends.^6,19^ Urban environments generally offer better access to education, contraception, and economic opportunities; all known fertility moderators.

The cross-national comparisons reflected underlying demographic and cultural realities revealing how union trajectories intersect with male fertility. The finding that South African men in multiple unions exhibit relatively high fertility, despite the country’s overall lower fertility levels and higher proportion of never-married men, reflects compositional effects. While national fertility is suppressed by delayed unions and higher non-marriage rates, the subset of men who enter multiple unions may represent a select group with distinct reproductive behaviours, including higher partner turnover and cumulative paternity.^35,37,51^ In contrast, Uganda presents a pattern where high fertility among men in multiple unions aligns with the country’s overall elevated fertility levels and earlier union formation, suggesting that such unions are more normative and socially embedded, thereby reinforcing widespread high cumulative paternity.^21,29,52^ Nigeria occupies an intermediate position, where relatively high fertility among men in multiple unions reflects the combined influence of rooted pronatalist norms and socioeconomic diversity.^23,25,27^ This indicates that multiple-union fertility in Nigeria is neither as selective as in South Africa nor as universally normative as in Uganda, but operates within a mixed demographic and socio-cultural context.^4,16,44^ South Africa’s lower male fertility also aligns with its advanced demographic transition and higher levels of contraceptive access and union instability.^51,53^ Uganda’s higher fertility is also likely associated with pronatalist norms and persistent barriers to male contraceptive engagement.^52,54^ In sum, the findings highlight how male fertility in Africa is predicted by complex intersections of marital trajectories, economic resources, and social expectations. They also call for more nuanced, male-inclusive reproductive health strategies that address both structural and cultural determinants.

### Implications for health and social policy and research

The findings from this study carry important implications for both population policy and future research in SSA. First, the clear association between marital union history and male fertility highlights the need for inclusive FP policies that actively target men, especially those in multiple or serial unions. Current reproductive health interventions are often female-centred, overlooking men’s union trajectories and their cumulative effect on fertility. Engaging men as active stakeholders in fertility decision-making, particularly in contexts of polygyny, remarriage, or partner concurrency, is essential for promoting responsible parenthood and reducing unplanned births. Second, the study emphasizes the role of socioeconomic inequality in shaping fertility outcomes, with poorer men in fewer or no unions experiencing lower fertility, while wealthier multi-union men maintain high fertility regardless of class. This suggests that fertility differentials are not only associated with access to resources but also by social norms and marital opportunities. Policymakers must therefore address the structural enablers of male union formation and reproductive behaviour, including access to employment, education, and social mobility, particularly for disadvantaged men.

Third, given the significant fertility disparities across urban and rural areas and across countries, reproductive health policies must be decentralized and culturally tailored. For example, the relatively lower fertility in South Africa reflects not only service access but also differing marital and reproductive norms. Interventions must be localized and context-specific, especially in high-fertility settings like Uganda, where male involvement in FP is often limited by gender norms and misconceptions. Fourth, the weak but statistically significant association between FP method use and fertility among men indicates a gap in FP uptake, especially among men using traditional methods. Governments and development partners should increase investment in male-centred FP education and service delivery, with a focus on improving awareness, accessibility, and gender-transformative approaches that challenge male dominance and fertility entitlement.

On implications for future research, this study reveals the importance of conceptualizing male fertility as both a demographic outcome and a social construct. It illustrates how this is influenced by marital pathways, economic class, and gendered opportunity. Future research should adopt longitudinal and qualitative designs to explore how men’s fertility intentions evolve over time and across unions, particularly in light of economic precarity, union instability, and migration. Moreover, while this study used robust nationally representative IPUMS-DHS datasets, future work should incorporate life history narratives and community-level variables to deepen understanding of how men navigate paternity across different social and institutional contexts. There is also a pressing need to reframe fertility studies to include male autonomy, union complexity, and intergenerational expectations, rather than treating male fertility as derivative of female outcomes. This includes investigating norms around ideal family size, legitimacy of children across unions, and male involvement in childrearing, all of which have implications for gender equality, child wellbeing, and population dynamics.

### Strengths and limitations

A major strength of this study lies in its use of harmonized, nationally representative IPUMS-DHS datasets from three diverse SSA countries (Nigeria, South Africa, and Uganda). This allows for a robust cross-national comparative analysis of male fertility. The large sample size (*N* = 20,587) enhances the statistical power and generalizability of the findings to wider male populations in similar contexts. Second, the study makes a unique contribution by focusing on male fertility patterns. This is an often-under-explored aspect of demographic research in Africa, particularly from the perspective of union history and household wealth positioning. By incorporating a marital union group × wealth interaction, the study reveals nuanced dynamics that go beyond simple additive effects. It highlights how marital history and household wealth interact to predict reproductive outcomes. Moreover, the application of GLMs with control for a wide range of covariates, including education, age at first union, FP use, employment, residence, and country, enhances the internal validity of the analysis and provides a comprehensive understanding of the predictors of male fertility.

Despite these strengths, this study is not without limitations. First, the cross-sectional design limits the ability to make causal inferences about the relationships between union status, wealth, and fertility. The observed associations represent snapshots in time and do not capture the temporal ordering of unions and childbearing. Second, the use of self-reported fertility data may be subject to underreporting, especially among men who are not co-residing with their biological children or are unaware of all their paternity history. This is particularly relevant for men with multiple unions or non-marital births and could lead to potential measurement bias. Third, while IPUMS-DHS provides harmonized variables, country-level differences in survey design and question phrasing may introduce comparability challenges, particularly regarding union definitions and fertility recall. Although country was controlled for in the models, unobserved contextual differences may still influence outcomes. Fourth, the analysis did not account for community- or partner-level variables, such as women’s fertility preferences, contraceptive negotiation, or cultural norms around polygyny and remarriage. These omitted factors may mediate or moderate the relationships observed and should be explored in future research. Also, the categorization of union status into never married, one union, and multiple unions does not distinguish between current marital states (e.g., currently married, divorced, separated, or cohabiting), which may mask differences in fertility trajectories across these groups. To address these limitations, the study applied multivariate modelling techniques (GLM) to control for a broad range of covariates, reducing confounding and enhancing the internal validity of the associations. Additionally, by using harmonized IPUMS-DHS data and controlling for country-level effects, the analysis mitigated potential comparability issues across national contexts. While causality cannot be established, the study offers robust, policy-relevant insights by identifying consistent patterns across large, diverse male populations.

## Conclusion

This study provides timely and important insights into the politics of paternity in SSA. It reveals how male fertility is not only a biological outcome but also a product of marital trajectories, household wealth (as an indicator of socioeconomic position), and structural opportunity. By demonstrating that men in multiple unions maintain high fertility irrespective of wealth status, while those in fewer or no unions experience fertility more closely tied to socioeconomic position, this study challenges simplistic assumptions about male reproductive behaviour and highlights the intersectional dynamics of union formation and fatherhood. The findings are urgent and policy-relevant in the context of Africa’s rapidly growing population, rising youth unemployment, persistent gender inequities, and overstretched health and social systems. If unaddressed, the continued marginalization of men in fertility and FP programmes could perpetuate gender-blind policies, reinforce unplanned population growth, and heighten the intergenerational transmission of poverty, particularly among men in lower household wealth groups and those with limited union exposure.

Moreover, failure to integrate men, especially those in polygynous or serial partnerships, into reproductive health policies risks undermining national efforts to achieve demographic transition, improve maternal and child health, and reduce inequalities in fertility outcomes. As such, the study calls for urgent, targeted, and male-inclusive interventions that recognize the social and structural dimensions of paternity. These should promote equitable reproductive health responsibilities, challenge harmful masculinities, and ensure that fertility and FP policies are responsive to the diverse realities of men’s lives across the continent. Without timely action, the continent may face a future where high male fertility persists as an unchecked driver of population pressure, socio-economic fragmentation, and deepened inequality, particularly across different household wealth strata; reversing gains made in health, education, and sustainable development.

## Supplemental Material

sj-docx-1-reh-10.1177_26334941261464394 – Supplemental material for Household wealth, marriage, and male fertility in sub-Saharan Africa: a cross-sectional comparative study from three countries Supplemental material, sj-docx-1-reh-10.1177_26334941261464394 for Household wealth, marriage, and male fertility in sub-Saharan Africa: a cross-sectional comparative study from three countries by Turnwait Otu Michael in Therapeutic Advances in Reproductive Health

sj-docx-2-reh-10.1177_26334941261464394 – Supplemental material for Household wealth, marriage, and male fertility in sub-Saharan Africa: a cross-sectional comparative study from three countries Supplemental material, sj-docx-2-reh-10.1177_26334941261464394 for Household wealth, marriage, and male fertility in sub-Saharan Africa: a cross-sectional comparative study from three countries by Turnwait Otu Michael in Therapeutic Advances in Reproductive Health

## Declarations

**Ethics approval and consent to participate:** This study is based on publicly available, anonymized data collected with informed consent secured by the DHS Programme (ICF IRB FWA00000845). IPUMS-DHS ensures strict confidentiality protocols in line with global research standards in accordance with the principles of the Helsinki Declaration. No additional ethical clearance was required for this secondary analysis; however, all analyses adhered to ethical principles of data protection, responsible reporting, and contextual interpretation.

**Consent for publication:** Not applicable.

**Availability of data and materials:** The data used for this study are publicly available from the IPUMS-DHS. Interested researchers can freely register on the IPUMS-DHS website (https://www.idhsdata.org/idhs/) and request access to the datasets. The raw data used to produce this study are also available from the correspondence author upon reasonable request.*
