# Supplementary material for: Household wealth, marriage, and male fertility in sub-Saharan Africa: a cross-sectional comparative study from three countries
Source: Ther Adv Reprod Health. 2026 Sep 23;20:26334941261464394. doi: 10.1177/26334941261464394 (PMC13760128; doi:10.1177/26334941261464394)
Supplement: Supplementary file 1 [file sj-docx-1-reh-10.1177_26334941261464394.docx]

**Supplementary Table S1: Distribution of respondents across marital union status and household wealth quintiles (weighted frequencies)**

**Crosstabs**

| **Notes** | | |
| --- | --- | --- |
| Output Created | | 21-APR-2026 15:45:05 |
| Comments | |  |
| Input | Data | C:\Users\Dell\OneDrive - University of Johannesburg\Documents\DOCUMENTS\DHS DATA SETS (SUB SAHARAN AFRICA\IPUMS DHS MEN Data _ Politics of Paternity & Fertility (3 Countries - Nig, ZA, Uganda) Only.sav |
|  | Active Dataset | DataSet1 |
|  | Filter | <none> |
|  | Weight | Men's sample weight |
|  | Split File | <none> |
|  | N of Rows in Working Data File | 20565 |
| Missing Value Handling | Definition of Missing | User-defined missing values are treated as missing. |
|  | Cases Used | Statistics for each table are based on all the cases with valid data in the specified range(s) for all variables in each table. |
| Syntax | | CROSSTABS  /TABLES=UNION_GROUP BY WEALTHQMN  /FORMAT=AVALUE TABLES  /CELLS=COUNT ROW COLUMN  /COUNT ROUND CELL. |
| Resources | Processor Time | 00:00:00.05 |
|  | Elapsed Time | 00:00:00.04 |
|  | Dimensions Requested | 2 |
|  | Cells Available | 524245 |

[DataSet1] C:\Users\Dell\OneDrive - University of Johannesburg\Documents\DOCUMENTS\DHS DATA SETS (SUB SAHARAN AFRICA\IPUMS DHS MEN Data _ Politics of Paternity & Fertility (3 Countries - Nig, ZA, Uganda) Only.sav

| **Case Processing Summary** | | | | | | |
| --- | --- | --- | --- | --- | --- | --- |
|  | Cases | | | | | |
|  | Valid | | Missing | | Total | |
|  | N | Percent | N | Percent | N | Percent |
| UNION_GROUP REC (frm Marital Status & No of Unions) * Household wealth index in quintiles (from men's file) | 20587 | 100.0% | .438 | 0.0% | 20587.438 | 100.0% |

| **UNION_GROUP REC (frm Marital Status & No of Unions) * Household wealth index in quintiles (from men's file) Crosstabulation** | | | | | | | | |
| --- | --- | --- | --- | --- | --- | --- | --- | --- |
|  | | | Household wealth index in quintiles (from men's file) | | | | | Total |
|  |  |  | Poorest | Poorer | Middle | Richer | Richest |  |
| UNION_GROUP REC (frm Marital Status & No of Unions) | Never married | Count | 1579 | 1670 | 1887 | 2044 | 1972 | 9152 |
|  |  | % within UNION_GROUP REC (frm Marital Status & No of Unions) | 17.3% | 18.2% | 20.6% | 22.3% | 21.5% | 100.0% |
|  |  | % within Household wealth index in quintiles (from men's file) | 45.8% | 45.9% | 45.7% | 44.7% | 41.0% | 44.5% |
|  | One union | Count | 1441 | 1551 | 1840 | 2181 | 2527 | 9540 |
|  |  | % within UNION_GROUP REC (frm Marital Status & No of Unions) | 15.1% | 16.3% | 19.3% | 22.9% | 26.5% | 100.0% |
|  |  | % within Household wealth index in quintiles (from men's file) | 41.8% | 42.6% | 44.6% | 47.7% | 52.6% | 46.3% |
|  | More than one union | Count | 427 | 418 | 399 | 344 | 307 | 1895 |
|  |  | % within UNION_GROUP REC (frm Marital Status & No of Unions) | 22.5% | 22.1% | 21.1% | 18.2% | 16.2% | 100.0% |
|  |  | % within Household wealth index in quintiles (from men's file) | 12.4% | 11.5% | 9.7% | 7.5% | 6.4% | 9.2% |
| Total | | Count | 3447 | 3639 | 4126 | 4569 | 4806 | 20587 |
|  |  | % within UNION_GROUP REC (frm Marital Status & No of Unions) | 16.7% | 17.7% | 20.0% | 22.2% | 23.3% | 100.0% |
|  |  | % within Household wealth index in quintiles (from men's file) | 100.0% | 100.0% | 100.0% | 100.0% | 100.0% | 100.0% |
